# Climate Adaptation and Policy-Induced Inflation of Coastal Property Value

**DOI:** 10.1371/journal.pone.0121278

**Published:** 2015-03-25

**Authors:** Dylan E. McNamara, Sathya Gopalakrishnan, Martin D. Smith, A. Brad Murray

**Affiliations:** 1 Department of Physics and Physical Oceanography/Center for Marine Science, University of North Carolina Wilmington, Wilmington, NC, 28403-5606, USA; 2 Department of Agricultural, Environmental and Development Economics, The Ohio State University, Columbus, Ohio, 43210, USA; 3 Nicholas School of the Environment, Duke University, Durham, NC, 27708, USA; Potsdam Institute for Climate Impact Research, GERMANY

## Abstract

Human population density in the coastal zone and potential impacts of climate change underscore a growing conflict between coastal development and an encroaching shoreline. Rising sea-levels and increased storminess threaten to accelerate coastal erosion, while growing demand for coastal real estate encourages more spending to hold back the sea in spite of the shrinking federal budget for beach nourishment. As climatic drivers and federal policies for beach nourishment change, the evolution of coastline mitigation and property values is uncertain. We develop an empirically grounded, stochastic dynamic model coupling coastal property markets and shoreline evolution, including beach nourishment, and show that a large share of coastal property value reflects capitalized erosion control. The model is parameterized for coastal properties and physical forcing in North Carolina, U.S.A. and we conduct sensitivity analyses using property values spanning a wide range of sandy coastlines along the U.S. East Coast. The model shows that a sudden removal of federal nourishment subsidies, as has been proposed, could trigger a dramatic downward adjustment in coastal real estate, analogous to the bursting of a bubble. We find that the policy-induced inflation of property value grows with increased erosion from sea level rise or increased storminess, but the effect of background erosion is larger due to human behavioral feedbacks. Our results suggest that if nourishment is not a long-run strategy to manage eroding coastlines, a gradual removal is more likely to smooth the transition to more climate-resilient coastal communities.

## Introduction

In the United States, the coastline is now the most densely populated region in the country, and population density continues to increase [1]. The coastal region hosts significant economic activity and some of the highest property values in the country. The erosion of coastal land from frequent storms, chronic wave driven currents, and rising sea level threatens coastal economies.

**Fig 1:**
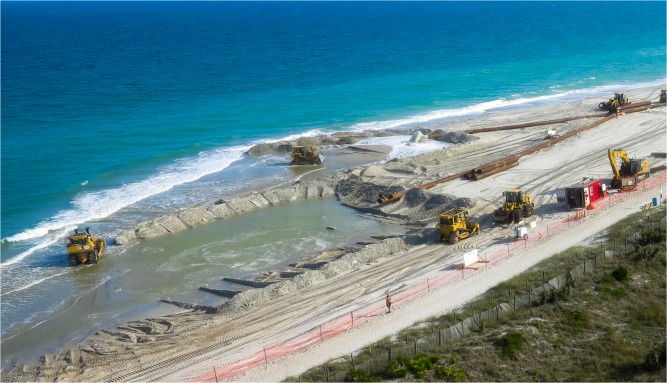
Beach Nourishment. Overhead view of a beach nourishment episode in Wrightsville Beach, NC during April of 2014.

To manage this vulnerability along sandy coastlines, humans nourish beaches—placing sand, typically from offshore sources, onto the beach to widen the beach and combat erosion (Fig. 1). Empirical data in the U.S. suggest that decisions to stabilize the shoreline through beach nourishment are made optimally, to maximize the value of recreation and storm protection benefits, at least from the perspective of local communities [2] (S1 File and S1 Fig.). Some locations along the U. S. East Coast have practiced beach nourishment for nearly 50 years [3]. Between 1995 and 2002, the U.S. federal government spent $787 million on beach nourishment and has historically subsidized two-thirds of total nourishment costs to coastal communities [4]. As humans alter physical coastal dynamics by temporarily reversing erosion, they influence real estate markets and, in turn, future beach management decisions [2, 5, 6]. Property values influence nourishment, nourishment influences beach width, and beach width feeds back on property values. In this way, human-occupied coastlines are strongly coupled systems, and policies that influence shoreline stabilization efforts become intrinsic drivers of economic value in the coastal zone.

Predictions of increased rates of sea level rise and changing storminess imply that coastal vulnerability will increase [7–9]. However, recent U.S. congressional sessions have considered cut backs in federal contributions toward beach nourishment (U.S. Senate Amendment #815). Because the coastal zone in many locations has evolved for decades as a coupled human-natural system, current outcomes reflect historical government subsidies. In a future of changing environmental forcing from sea level and storms, and potential policy changes such as decreased subsidies to nourishment, the configuration of coastal systems—and associated shoreline positions and real estate markets—may change dramatically.

We use a stochastic dynamic optimization model to explore the influence of storms, rising sea level, and changing management approaches on coastal property values. The optimal nourishment frequency for the beach determined by the model reflects a property owner’s point of view, taking physical forcing and property values into consideration. We represent the evolution of the beach as three components: chronic background erosion (e.g. from rising sea level or from alongshore sediment transport gradients), erosion that results as beaches and shorelines adjust to nourishment events, and erosion from punctuated storm events. The erosion from stochastic storm events is represented as a loss of the entire nourished portion of the beach that tends to occur during a hurricane [10]. Optimal beach nourishment occurs when financial capital is invested in natural capital (the beach fronting coastal properties) to maximize the value of coastal property. The model allows us to explore the impacts on nourishment frequencies and real estate values from distinct sources of climate forcing. We also use the dynamic optimization to investigate the diminution of property value from reducing the federal subsidy for beach nourishment. We compare property values with and without the typical subsidy of nourishment costs. We characterize the nourishment subsidy as driving a wedge between the traded value of a property and its underlying fundamental value. The wedge in coastal real estate is not simply a reflection of economics, but its occurrence and size reflect the environmental forcing regime and the coupling between coastal dynamics and coastal economies.

## Methods

We explore how the coupled physical-economic coastline depends upon changes in policy and physical forcing through a positive analysis in which managers follow optimal beach nourishment practice. While there is a large body of work on normative analysis and valuation studies of coastal economies [11–17], our goal, in contrast to previous efforts, is to quantify the current configuration of the coupled coastal system and then explore how that configuration will change as policy and physical forcing changes. Specifically, beach nourishment is modelled as an optimal rotation problem in which coastal managers choose a constant time interval between nourishment events to maximize net benefit [5]. Beach width (x) depends on linear background erosion (ϒ) and exponential decay (θ) of the nourished portion (μ) of the beach as it returns to the equilibrium profile. Every time beach nourishment is undertaken, the beach returns to its initial width (x_o_). Beach width at any given time (t) is characterized by:

$$x(t)=x_{o}-\gamma t-\mu x_{o}(1-e^{-\theta t}).$$ (1)

Benefits from wide beaches are capitalized in to coastal property values and total benefits for an interval T between two nourishment projects are:

$$B(T)=\int_{0}^{T}e^{-\delta t}\delta A\left(x{\left(t\right)}^{\beta }\right)dt$$ (2)

where A is the baseline value that captures the value of all property attributes except beach width, β reflects the capitalized value of beach width accounting for nourishment feedbacks (derived from previous empirical studies [2]), and δ is the discount rate for future benefits and costs. We assume that discount rate is the same as the capitalization rate to convert the stock value of the property to amenity flows. We hold the value of A and β fixed for subsidized and non-subsidized simulations. While it is possible that these values and hence property value could increase in a few select locations as subsidization policy was altered, such increase would necessarily be met with decreases in many other locations as property buyers sort across communities. In the absence of a spatially extended model for determining the spatial distribution of property values, we fix the value of A and β as a conservative estimate when quantifying property value reductions due to the loss of nourishment subsidies (declines in both A and β would exacerbate diminution of property value).

Nourishment costs include fixed costs (c) and variable costs (Φ) that depend on the amount of nourishment sand required to return the beach to its initial width:

$$\begin{array}{l} C(T)=c+\phi (x_{o}-x(T))\\ \;\;=c+\phi (\mu x_{o}(1-e^{-\theta T})+\gamma T) \end{array}$$ (3)

We extend the optimal rotation model above, which is rooted in forest economics, to include punctuated storm events. This extension follows the introduction of stochastic forest fires into optimal forest management [18]. We assume that hurricanes are Poisson distributed [19] and if a storm occurs at some time (τ) before the next nourishment period (T), then the benefits from nourishment accrue only to the moment the storm occurs. Let τ_1_, τ_2_, τ_3_, represent times between loss of nourished beach width (times between nourish events or storms). If storms are Poisson distributed and planned nourishment interval is denoted as T then the cumulative distribution function for the independent random variable τ_i_ is written as:

$$\mathrm{Pr}(\tau \leq t)=F(t)=\left\{\begin{array}{ccc} 1-e^{-\lambda t} & if & t<T\\ 1 & if & t\geq T \end{array}\right\}$$ (4)

This leads to a probability distribution function given by

$$\frac{d}{dt}F(t)=f(t)=\left\{\begin{array}{c} \lambda e^{-\lambda t}\\ e^{-\lambda t} \end{array}\right\}$$ (5)

With the possibility of a storm, the net benefits π (τ_k_) from the k^th^ nourishment episode are:

$$\pi (\tau_{k})=\left(\begin{array}{ccc} B(T)-C(T) & if & \tau_{k}=T\\ B(\tau )+B_{2}(T-\tau )-C(T) & if & \tau_{k}<T \end{array}\right)$$ (6)

If a storm occurs before the next nourishment (T), then benefits from replenishment (nourished portion of the beach) accrue only to the moment the storm occurs (τ _k_). After a storm, the nourished portion of the beach is lost and benefits accrue only from the remaining beach, which faces background linear erosion [10]. This representation of storms reflects the assumption that a significant departure from a nourishment schedule would require mobilizing funding and capital equipment as well as securing sand resources and permitting. Exceptions would occur for emergency stabilizations, such as filling new inlets in barrier islands.

The total expected present value of all future cost and revenue is

$$J(T)=E\left\{\sum_{k=0}^{\infty }e^{-\delta (\tau_{0}+\tau_{1}+\tau_{2}+\ldots \tau_{k})}\pi (\tau_{k+1})\right\}.$$ (7)

Because the random variables τ_k_ are independent, the expectation of the inner product in the summation can be split as

$$E\left\{e^{-\delta (\tau_{0}+\tau_{1}+\tau_{2}+\ldots \tau_{k})}\pi (\tau_{k+1})\right\}={\left(E\left\{e^{-\delta \tau }\right\}\right)}^{k}E\left\{\pi (\tau )\right\}.$$ (8)

Taking the first expectation in the above expression yields

$$\begin{array}{l} E\left\{e^{-\delta \tau }\right\}=\int_{0}^{\infty }e^{-\delta \tau }f(\tau )d\tau \\ \;\;\;=\frac{\lambda +\delta e^{-(\lambda +\delta )T}}{\lambda +\delta } \end{array}$$ (9)

Similarly, the second expectation from Eq. (8) gives

$$\begin{array}{l} E\left\{\pi (\tau )\right\}=\int_{0}^{T}\left[B(t)+B_{2}(T-t)-C(T)]\lambda e^{-\lambda t}dt+[B(T)-C(T)\right]e^{-\lambda T}\\ \;\;\;=\int_{0}^{T}\left[B(t)+B_{2}(T-t)]\lambda e^{-\lambda t}dt+C(T)(e^{-\lambda T}-1)+[B(T)-C(T)\right]e^{-\lambda T} \end{array}$$ (10)

and putting these results back into Eq. (7) results in

$$J(T)={\sum_{k=0}^{\infty }\left(\frac{\lambda +\delta e^{-(\lambda +\delta )T}}{\lambda +\delta }\right)}^{k}\left[\begin{array}{c} C(T)(e^{-\lambda T}-1)+(B(T)-C(T))e^{-\lambda T}\\ +\int_{0}^{T}[B(t)+B_{2}(T-t)]\lambda e^{-\lambda t}dt \end{array}\right]$$ (11)

Summing the infinite geometric series and cancelling common terms gives

$$J(T)=\left(\frac{\lambda +\delta }{\delta (1-e^{-(\lambda +\delta )T})}\right)\left[B(T)e^{-\lambda T}-C(T)+\int_{0}^{T}[B(t)+B_{2}(T-t)]\lambda e^{-\lambda t}dt\right]$$ (12)

Now we take the derivative of Eq. (12) and set it to zero to give an expression that can be solved for the T that maximizes expected net benefits:

$$\begin{array}{c} \frac{[B'(T)e^{-\lambda T}-C'(T)](\lambda +\delta )(1-e^{-(\lambda +\delta )T})-[B(T)e^{-\lambda T}-C(T)]{\left(\lambda +\delta \right)}^{2}e^{-(\lambda +\delta )T}}{\delta {\left(1-e^{-(\lambda +\delta )T}\right)}^{2}}-\\ \frac{\lambda {\left(\lambda +\delta \right)}^{2}e^{-(\lambda +\delta )T}\int_{0}^{T}B(t)e^{-\lambda t}dt}{\delta {\left(1-e^{-(\lambda +\delta )T}\right)}^{2}}=0 \end{array}$$ (13)

The interval T that solves Eq. (13) is the optimal time between nourishments, accounting for the expectation of storm events. Note that if λ is set to 0, the above expression reduces to the simpler non-stochastic rotation problem [5]. We use an unconstrained nonlinear optimization procedure to numerically determine the optimal rotation. Integrals are evaluated with an adaptive Simpson quadrature routine with storms return intervals drawn from a Poisson distribution.

## Results

**Fig 2:**
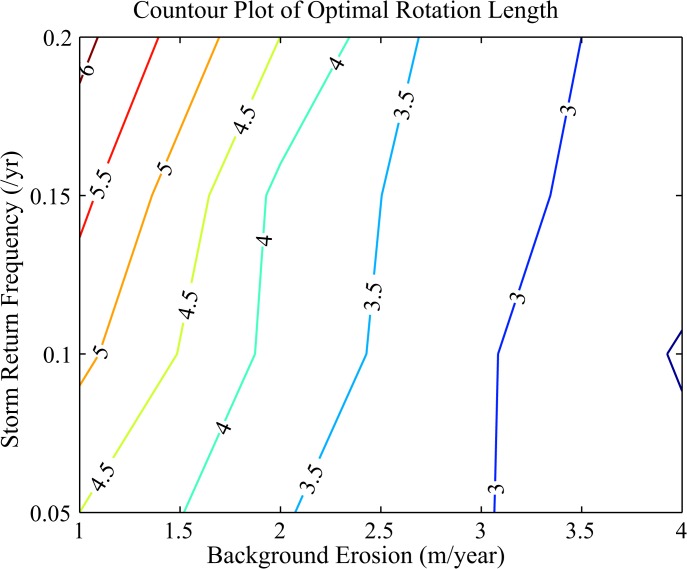
Optimal Intervals in Nourishment Events. Contours for the optimal time in years between nourishment events plotted against the mean background erosion rate (m/yr) and the mean number of storms per year. Historically, the background erosion rate for North Carolina is near 1 m/yr (21). The return period for hurricanes in this region ranges between 8 and 20 years (22).

We choose economic parameters for the analysis to mimic a typical town along the coast of North Carolina (S1 File) and explore optimal nourishment over a range of environmental forcing conditions associated with changing storminess and changing background erosion rate. Numerical solutions for optimal nourishment intervals show that as background erosion increases the optimal period between beach nourishment decreases (Fig. 2). This intuitive result reflects the need to replace beach more frequently as the beach erodes more rapidly. This result occurs despite the associated increased costs because background erosion accrues linearly with time, so discounted near-term benefits can outweigh costs incurred in the future. Counter-intuitively, the optimal nourishment interval increases as storms become more frequent (Fig. 2). The result parallels the introduction of stochastic forest fires into rotational forestry [18], with some subtle differences. In forest rotations, greater fire frequency shortens the rotation and reinforces the effect of discounting (discounting also shrinks the rotation length). In our case, greater storm frequency works in the opposite direction of discounting by increasing rotation length. When forest fires arrive more often, waiting longer to cut the forest exposes more value to risk, so shrinking the rotation is optimal. When storms arrive more often on the coast, waiting longer to nourish exposes less value to risk; nourishment projects (and the associated capital invested in them) are not destroyed as often. For a given nourishment interval, the expected benefits from nourishment decrease as storminess increases the probability of losing natural capital earlier in the nourishment cycle. Therefore, it becomes more advantageous to increase the time between nourishment to reduce continued loss of recently nourished beach.

**Fig 3:**
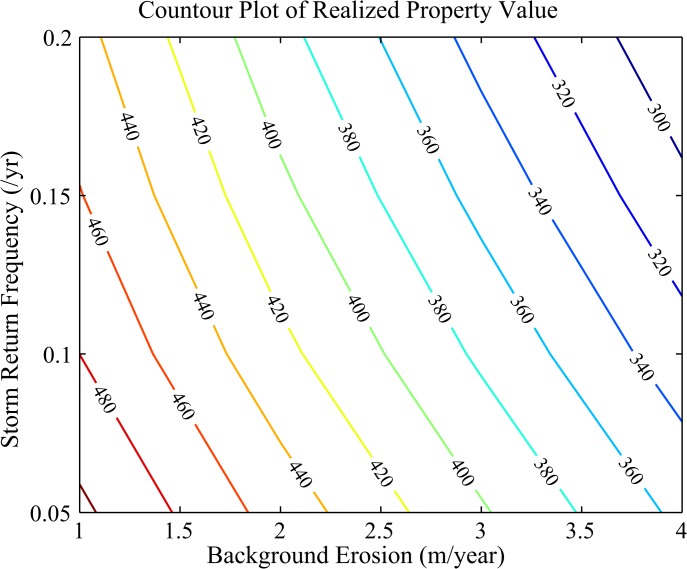
Property Values at Optimal Nourishment. Contours of discounted long-term net benefits (x$1000), or property value, at the optimal nourishment schedule plotted against the mean background erosion rate (m/yr) and the mean number of storms per year.

Once the optimal nourishment interval is determined, the realized property value is found by calculating the total discounted net benefits from beach width at the optimal nourishment interval and a given baseline property value that captures all other attributes except width (see Eq. 6 in Methods). We find that the property value decreases as the rate of background erosion (e.g. from sea level rise) increases and as the rate of storminess increases (Fig. 3). Both trends are expected since increasing background erosion and increasing erosion from storms reduces the amount of natural capital.

**Fig 4:**
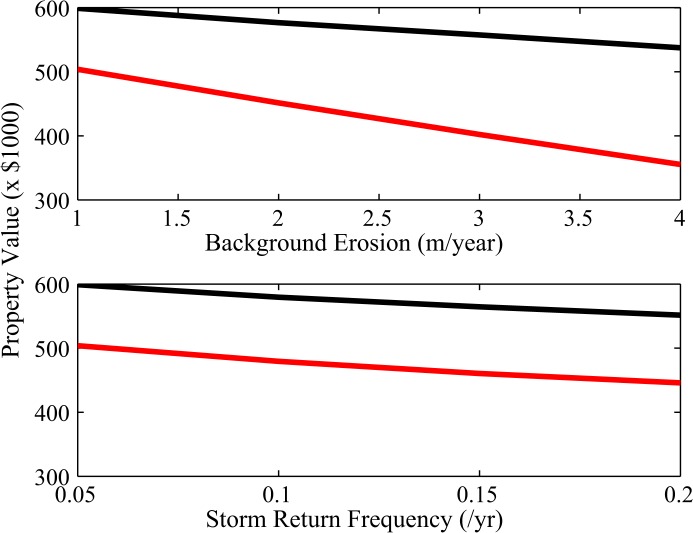
Current Inflation in Property Value with Nourishment Subsidy. Top panel shows property value versus background erosion rate (m/yr) when the mean number of storms per year is 0.05. Bottom panel shows property value versus mean number of storms per year when the background erosion rate is 1 m/yr. The black lines correspond to simulations where 66% of nourishment costs are subsidized by the federal government and the red lines are for simulations where costs are paid entirely by the local community. Arrows indicate the gap in property value due to a switch from subsidized to unsubsidized nourishment.

Historically, the federal government has subsidized nourishment costs by paying approximately 66% of the total cost [4, 20]. We investigate the impact of losing this subsidy by comparing net benefits, or property value, when the entire cost to nourish is paid by the local community and when the nourishment cost paid by the community is only 34% of actual cost. Holding the storm climate at the level of hurricanes returning every 20 years, the decrease in property value from the loss of nourishment cost subsidy approaches 34% as erosion from rising sea level reaches 4 m/yr (Fig. 4). The nourishment subsidy thus results in inflated values of oceanfront properties as they capitalize the total benefits from nourishment but pay only a share of the costs. The wedge between the realized value of an oceanfront property and the fundamental value of net benefits from the bundle of services it provides is akin to a bubble in the coastal real estate market resulting from the coupled dynamics of the geomorphology and economic policy. When the erosion rate remains at 1 m/yr the size of the wedge only varies from 16% to 19% as hurricane return periods shorten from every 20 years to every 5 years (Fig. 4).

**Fig 5:**
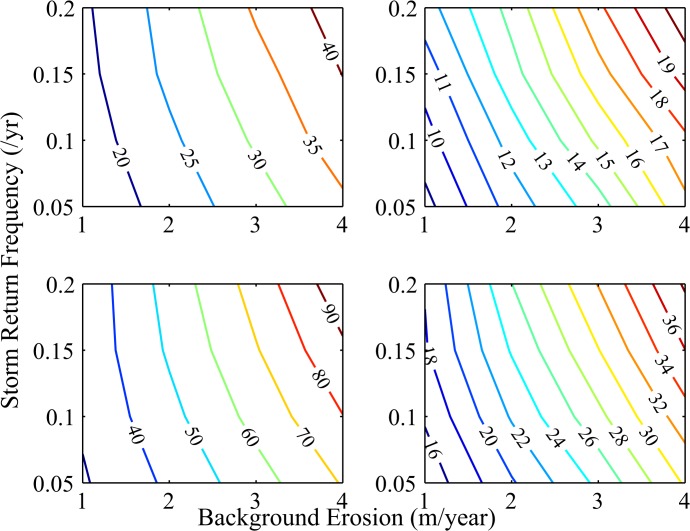
Reductions in Property Value with Loss of Subsidy over a Range of Communities and Nourishment Costs. Contours of the percent reduction in property value versus background erosion rate (m/yr) and mean number of storms per year for four cases: Top left panel—high baseline property value (parameter A = $200,000) and high nourishment sand cost ($20/m^3^). Top right panel—high baseline property value (parameter A = $200,000) and low nourishment sand cost ($10/m^3^). Bottom left panel—low baseline property value (parameter A = $100,000) and high nourishment sand cost ($20/m^3^). Bottom right panel—low baseline property value (parameter A = $100,000) and low nourishment sand cost ($10/m^3^).

To explore heterogeneity in baseline property values, we compare our analysis with one using economic parameters representative of a higher property value location (Fig. 5). Together, these two cases span a range of property value that encompasses most of the towns in coastal New Jersey and North Carolina (S1 File). When baseline property values are higher, the size of the property value wedge is reduced, reaching about 21% for background erosion rates of 4 m/yr and hurricane return periods of 5 years, whereas the wedge reaches nearly 43% for the lower property value location for these erosion and storm scenarios.

We also explore how an increasing cost of acquiring nourishment sand impacts the size of the policy-induced wedge in value for the cases of high and low property value (Fig. 5). As beach compatible sand becomes more difficult to locate [21], it is expected that nourishment sand will become more expensive. We find that doubling the cost of sand leads to a larger wedge—i.e. more policy-induced diminution in value. In the worst case, a low property value home subject to erosion rates of 4m/yr and a hurricane return period of 5 years would suffer a 95% drop in property value should the subsidy for nourishing be removed. The intuition is that the cost of nourishing a given length of the shoreline does not vary across high and low value properties, so the subsidy is a larger share of the total for low property values. When costs rise, this already large share increases.

If the nourishment subsidy were to disappear at present, based on historical records for mean background erosion rate [22] and hurricane return periods [23], and assuming nourishment sand costs are at historical values, the current inflation in coastal North Carolina and New Jersey would be nearly 9% for high property value locations and about 16% for low property value locations. While these estimates reflect mean environmental conditions, some locations in North Carolina and New Jersey currently suffer from erosion rates of 4 m/yr [22]. In these towns, a removal of nourishment subsidy could erode fundamental property values nearly 17% and 34% for high and low property value regions respectively.

## Discussion

The optimization model used here is parameterized based on existing empirical estimates of the value of beach width in North Carolina, which shows that, accounting for nourishment feedbacks improves the match between model predictions of nourishment intervals and observed nourishment intervals [2]. This work extends that comparison (S1 File and S1 Fig.) by adding stochastic storms to the physical dynamics and removing historical nourishment episodes that occurred due to placement of sand left over from nearby inlet channel dredging. Such comparisons of model outputs with empirical data provide some measure of confidence that the modeling assumptions used here reflect the dynamics of historical beach nourishment decisions. The removal of the nourishment subsidy, and the subsequent imposition of nourishment costs on oceanfront homeowners has not happened and therefore our simulations exploring the impacts of such a forcing change represent predictions of future behavior if forcings change in that manner.

Our analysis abstracts away from political process dynamics and funding mechanisms that vary across localities and focuses on physical dynamics and economic value as the principal drivers of beach nourishment. Other institutional factors could introduce more dynamics, but despite our abstraction we are able to explain variation in observed nourishment intervals of this coupled system (S1 Fig).

Reductions in property value from increasing erosion and storms, as our results show, are expected to play out over many years to decades as the natural system slowly changes. In contrast, if the federal government were to remove subsidies for beach nourishment causing local municipalities to pay the entire cost, the intrinsic value of coastal property would instantly decline, by as much as 34% in some locations. This highlights how vulnerability of many coastal towns is not only attributable to exogenous shocks in the form of damage from large storm events, which garner much of society’s attention, but also reflects the intertwined nature of coastal property value, coastline behavior, and coastal management policy.

Although our model predicts a sizable policy-induced inflation in coastal real estate markets, it is neither a call for increasing subsidies nor a call for removing them. Subsidies for beach nourishment are a part of a broader suite of policies that manage coastal hazards. These other policies, including subsidized flood insurance and disaster relief, expose public funds to tremendous risk [24], and what seem like very costly adaptive measures can be justified by avoided expected storm damage [25, 26]. It may be that subsidies to beach nourishment, by reducing demand for disaster relief, constitute a more efficient use of federal dollars relative to other subsidized programs. Whether or not nourishment is an efficient use of public funds, if the subsidy were removed suddenly, it could have dramatic consequences, including a crash in coastal property values in regions relying heavily on subsidized nourishment and associated drops in local property tax revenues that fund local public goods such as schools and infrastructure. As such, we recommend a cautious approach to changing subsidies for beach nourishment. If nourishment is not part of a long-run strategy to manage eroding coastlines, a gradual phase-out is more likely to smooth the transition to more climate-resilient coastal communities.

## Supporting Information

S1 File A detailed description of the numerical configuration of the optimization model for comparing model output with empirical data for beach nourishment. The physical [22,23] and economic [2,5,27] model parameters are set to mimic conditions in four coastal cities in North Carolina.
(DOC)

S1 Fig Comparison of Empirical and Modeled Nourishment. Historical (black dots) and modeled nourishment (red circles) intervals for four coastal locations: Wrightsville Beach (WB), Carolina Beach (CB), Emerald Isle (EI) and Kure Beach (KB). Error bars correspond to the 95% confidence intervals.
(EPS)
