# Supplementary material for: Climate Adaptation and Policy-Induced Inflation of Coastal Property Value
Source: PLoS One. 2015 Mar 25;10(3):e0121278. doi: 10.1371/journal.pone.0121278 (PMC4373792; doi:10.1371/journal.pone.0121278)
Supplement: S1 File — The physical [22,23] and economic [2,5,27] model parameters are set to mimic conditions in four coastal cities in North Carolina. (DOC) [file pone.0121278.s001.doc]

Climate Adaptation and Policy-Induced Inflation of Coastal Property Value

**SUPPORTING INFORMATION**

Parameter values used in the numerical analysis are based on previous studies [2, 5]. Nourished beach width, , is 50m, which is similar to the average width observed in an empirical study of ten beach towns in North Carolina [2]. The portion of the nourished beach that decays exponentially, , is set to thirty five percent [5], and the nourishment decay rate, , is set to 0.10 [5]. Punctuated erosion events in the model are represented by category 1 or higher hurricanes with return intervals modeled as a Poisson process [19]. The discount rate, , for the economic analysis is set to 0.06 [5]. Nourishment costs consist of fixed and variable costs. We assume fixed costs, , of capital equipment to be $1 million normalized over 100 ocean front properties [2, 5]. Variable costs, , are assumed to be $2000 per home per cross-shore meter of beach build-out. This assumes sand costs are $10 per m^3^ [2, 5, 27], there are approximately 50 homes per kilometer of beach alongshore [5], and that nourishment sand spreads to a depth of 10m – approximately the shoreface closure depth. For simulations representing the lower property value regions we assume a baseline property value, A, that captures the value of all housing attributes excluding beach width to be $100,000 [2]. The hedonic value of beach width, , is 0.5 [2]. For higher property value locations we change the value of the baseline property to $200,000 [2].

The range of baseline property vales used in the numerical analysis is representative of locations along the U.S. Atlantic coast based on sales transactions data from local tax assessors’ offices. The price of an average coastal property in New Jersey is $573,384, and locations with highest property values such as Sea Girt and Long beach, coastal property values are close to $1,500,000. Property values in North Carolina, based on an empirical study [2] range from $275,000 in Carolina beach to nearly $800,000 in Wrightsville beach. We isolate baseline property values, excluding the value of beach width accounting for the nourishment feedback, based on previous studies [2, 5]. For example, if the value of an oceanfront property in Wrightsville beach is $1,500,000, and the beach is 50m wide, baseline value can be determined by: P = A(x)^β^, which implies A = P/((x)^β^,) = 1500000/(50^0.5) = $212000. Similarly, the baseline value of an average property in New Jersey is 573,384//(50^0.5) = $92275. We therefore use $100,000 and $200,000 as baseline values for low- and high-value towns.

We numerically solve for optimal nourishment intervals with economic [2] and physical [22, 23] model parameters that match 4 coastal towns (see Table below). These intervals are compared with historical nourishment intervals (Fig. S1) and the numerical results fall within the 95% confidence intervals of the measured values, except for Kure Beach which has nourished at fixed 3 year intervals since inception of nourishment practices. The historical record of nourishments is taken from the Program for the Study of Developed Shorelines ([http://psds.wcu.edu](http://psds.wcu.edu/)) where we use only nourishment episodes since 1980 so as to avoid large changes in parameter settings, which are fixed in the numerical solution. Furthermore we neglect nourishment events in the historical record that were for the navigation purposes – dredging a nearby coastal inlet and placing sand on the beachfront.

| **Location** | **Baseline Property Value** (A - $ m^-0.5^) | **Mean Erosion Rate** (γ m yr^-1^ ) | **Storm Return Interval** (λ - yr^-1^) |
| --- | --- | --- | --- |
| Wrightsville Beach (WB) | 161 | 1.0 | 0.1 |
| Carolina Beach (CB) | 73 | 1.0 | 0.1 |
| Kure Beach (KB) | 122 | 1.0 | 0.1 |
| Emerald Isle (EI) | 119 | 0.5 | 0.125 |

Parameter values used for numerical solution of optimal nourishment intervals.
